# Outpatient opioid dispensing during the 2024 floods in Southern Brazil: a population-based ecological time-series analysis

**DOI:** 10.1016/j.lana.2026.101646

**Published:** 2026-09-22

**Authors:** Enzo Fabian Frachia, Juliana Vargas Campiol, Santiago Madeira Diefenthaeler, Felipe Ornell, Simone Hauck, Flavio Kapczinski, Juliana Nichterwitz Scherer

**Affiliations:** a Programa de Pós-Graduação em Saúde Coletiva, Universidade do Vale do Rio dos Sinos (UNISINOS), São Leopoldo, RS, Brazil; b Universidade Federal do Rio Grande do Sul (UFRGS), Porto Alegre, RS, Brazil; c Programa de Pós-Graduação em Psiquiatria e Ciências do Comportamento, Universidade Federal do Rio Grande do Sul (UFRGS), Porto Alegre, RS, Brazil

**Keywords:** Opioid analgesics, Climate-related disasters, Ecologicaltime-series analysis, Outpatient care, Public health systems

## Abstract

**Background:**

Climate-related disasters can disrupt health-care delivery and treatment continuity. Evidence on their effects on opioid dispensing in middle-income countries remains scarce. We assessed whether the May 2024 floods were associated with changes in outpatient opioid dispensing in Rio Grande do Sul, Brazil.

**Methods:**

We conducted a retrospective ecological time-series study using official Brazilian Ministry of Health data on codeine, morphine, and methadone dispensations from 2015 to 2025. Monthly rates per 10,000 inhabitants were modelled with quadratic secular and seasonal terms, month-specific effects from May 2024 to May 2025, and Newey–West standard errors. Analyses were stratified by opioid and age, applied to Minas Gerais as a comparison state, and repeated after excluding 2020–2021.

**Findings:**

Among 131 states-months analysed, May 2024 was associated with an abrupt reduction of 68.86 dispensed units per 10,000 inhabitants relative to the counterfactual (95% CI −73.57 to −64.15; p < 0.001), followed by a smaller reduction in June (−14.81 per 10,000 inhabitants; 95% CI −21.12 to −8.51; p < 0.001). Females accounted for 63.6% of all dispensed units. From July to September, dispensing no longer differed significantly from expected levels, followed by an uneven recovery with intermittent above-expected increases. Disruption was most prolonged for morphine. The immediate decline remained after excluding the COVID-19 period.

**Interpretation:**

The abrupt, transient decline suggests that the floods disrupted the health-care network and continuity of access to controlled medicines. Although aggregate dispensing recovered, this does not ensure that therapeutic continuity was fully restored for all patients. Disaster preparedness and response should therefore prioritise health-system resilience capable of preserving essential treatments during climate-related emergencies.

**Funding:**

Fundação de Amparo à Pesquisa do Estado do Rio Grande do Sul (FAPERGS), Conselho Nacional de Desenvolvimento Científico e Tecnológico (CNPq), and Instituto Nacional de Ciência e Tecnologia em Saúde Mental Digital (INCT-SMD).

**Research in context**

**Evidence before this study**

We searched PubMed, Scopus, and Web of Science from Jan 1, 2000, to Dec 31, 2024, using combinations of the terms “opioid dispensing”, “opioid prescribing”, “opioid analgesics”, “medication access”, “natural disaster”, “flood”, “hurricane”, “cyclone”, “climate-related disaster”, “health system disruption”, “longitudinal analysis”, “ecological study”, and “time series”. We also screened the reference lists of relevant articles. No language restrictions were applied.

Previous studies have documented disruptions in health-care delivery following hurricanes, cyclones, and other extreme weather events, including reduced access to chronic disease management, hypertension care, and maternal services. Some evidence from high-income countries has examined access to medications for opioid use disorder following disasters.

However, we identified no population-based longitudinal study assessing temporal changes in outpatient opioid analgesic dispensing associated with extreme flooding in Latin America or other middle-income settings. Existing evidence has largely focused on high-income countries, general health-service utilisation, or treatment programmes for opioid use disorder, rather than the broader dispensing of opioid analgesics within a universal public health system.

**Added value of this study**

Using population-based administrative data from Brazil’s Unified Health System, this study provides, to our knowledge, the first non-parametric ecological time-series assessment of changes in outpatient opioid dispensing during a major flood in Latin America. We identified a marked reduction in total opioid dispensing in May 2024, which remained significant in the following month, followed by relative stabilisation and an uneven recovery with intermittent above-expected dispensing. The acute decline was observed across codeine, morphine, and methadone and across all age groups, although the magnitude, duration, and recovery trajectories differed substantially between opioid agents and demographic strata. The immediate reduction remained evident after excluding the COVID-19 period, and the contextual comparison with Minas Gerais showed no comparable contemporaneous decline in total dispensing, providing cautious support for a regionally specific disruption.

**Implications of all the available evidence**

Extreme flooding may abruptly disrupt the recorded delivery of controlled medicines, even within a large universal public health system. The heterogeneous recovery observed across opioid agents and age groups indicates that restoration of aggregate dispensing activity does not necessarily represent the recovery of individual treatment continuity. Climate-resilience planning should therefore protect pharmaceutical supply chains, dispensing and authorisation pathways, administrative reporting systems, and access for patients dependent on continuous treatment. Preparedness strategies should include contingency stocks, alternative dispensing routes, emergency prescribing and authorisation mechanisms, interoperable health-information systems, and proactive identification and follow-up of patients whose treatment cannot be safely interrupted. These measures may be particularly relevant for health systems in low- and middle income settings that are increasingly exposed to severe climate-related events.

## Introduction

Chronic pain represents a major global public health challenge, affecting quality of life, functional capacity, and health system utilization. A global study across 52 countries documented an average pain prevalence of 27.5%, with substantial cross-national variation.^1^ In Brazil, chronic pain affects 35.7% of adults,^2^ positioning the country among those with an elevated pain burden. Opioid analgesics remain essential for managing moderate-to-severe pain when non-opioid therapies are insufficient,^3^ yet access in Latin America remains limited despite high pain prevalence.^4^ In Brazil, recent population-level evidence indicates a rapid expansion in exposure to opioid analgesics. According to the Third Brazilian National Survey on Alcohol and Drugs (LENAD III), the largest national survey on alcohol and drug use, the proportion of the population reporting lifetime use of opioid analgesics increased ninefold over a decade, from 0.8% in 2012 to 7.6% in 2023.^5^ This trend is particularly relevant because opioids represent a rare trifecta, being used for legitimate pain management, functioning as illicit drugs subject to misuse, and carrying broader implications for public safety and security. In this context, outpatient opioid prescribing requires careful monitoring due to risks of dependence and inappropriate use, making prescribing patterns important indicators of pharmaceutical governance that may be compromised under exceptional circumstances.^6^^,^^7^

Extreme climate events have emerged as critical determinants of health, disrupting service delivery, compromising medication supply chains, and interrupting continuity of care.^8^ Recent disasters have exposed vulnerabilities in pharmaceutical supply chains with cascading effects on essential medication access.^9^ Climate-related disasters disproportionately affect vulnerable populations and deepen inequities in healthcare access.^8^ Analyses of routinely collected health-service data have documented substantial disruptions following cyclones and hurricanes,^10^^,^^11^ illustrating the value of temporal data for characterising disaster-related changes in health-care delivery.

Opioid prescribing occupies a unique position at the intersection of pain management and health system resilience. Medication interruptions following disasters particularly affect patients dependent on continuous pharmacotherapy,^12^ with opioid access disruptions potentially causing untreated pain, physiological withdrawal related to abrupt treatment interruption, and health deterioration.^13^ Conversely, theoretical and empirical frameworks suggest that disasters may increase substance use through psychological mechanisms, including heightened emotional distress and self-medication behaviours.^14^ Accordingly, monitoring the dispensing of medications with potential for misuse following such events may serve as an indirect estimate of changes in substance use and overall health system functioning. Despite growing climate–health research, empirical evidence on how extreme events affect outpatient opioid dispensing remains scarce in low- and middle-income countries. In this context, Brazil offers a particularly informative setting: a continental-sized country with a universal public health system that provides care free at the point of use,^15^ where approximately 75% of the population lacks private health insurance. As a result, dispensing patterns within the public system capture responses to system shocks and may provide insights relevant to other countries with similar health system structures and access constraints.

In May 2024, Rio Grande do Sul, Brazil, experienced unprecedented flooding that severely disrupted infrastructure and health services across most municipalities.^16^ The flood affected 476 of 496 municipalities, directly impacting 2,398,255 people (≈22% of the state population), with 581,643 displaced and 80,826 sheltered, producing a large-scale collective trauma and placing sustained pressure on health services and community networks. Within Brazil’s Unified Health System (SUS), this event constitutes a natural experiment to examine the impacts of large-scale flooding on outpatient opioid dispensing. The SUS provides comprehensive pharmaceutical coverage yet faces persistent access challenges and supply chain vulnerabilities, particularly during emergencies.^16^ This study analysed trends in outpatient dispensing of codeine, methadone, and morphine, the primary SUS-formulary opioid analgesics, in Rio Grande do Sul between 2015 and 2025, assessing whether 2024 floods were associated with abrupt dispensing changes. Using non-parametrictime-series analysis of population-based administrative data, we examine changes in recorded pharmaceutical dispensing in the context of climate-related disasters within a universal health system. Evidence on the association between flooding events and opioid dispensing patterns in Latin America remains scarce, and this study contributes to filling that gap.

## Methods

### Study design

This retrospective ecological time-series study used population-based administrative data to examine changes in outpatient opioid dispensing before, during, and after an extreme flood event. A non-parametric analytic approach, allowing the effect of the flood to be estimated independently for each post-event month, was selected as the primary analytical strategy given its suitability for quasi-experimental evaluation of exogenous shocks affecting health systems at the population level, without imposing an a priori functional form on the pace or shape of the post-event trajectory. This design allows for the assessment of both immediate changes in outcomes and shifts in underlying trends following a clearly defined intervention or event. This study was designed and reported in accordance with the STROBE (Strengthening the Reporting of Observational Studies in Epidemiology) statement for observational research.^17^

### Data source and population

Data were extracted from the Ambulatory Care Information System (Sistema de Informações Ambulatoriais — SIA-SUS), a comprehensive administrative database managed by the Department of Informatics of the Brazilian Unified Health System (DATASUS). The SIA-SUS captures outpatient procedures and records of dispensed units reimbursed by the public health system and is widely used for pharmacoepidemiological surveillance and health services research in Brazil.^18^ Data extraction was performed using the microdatasus package in the R programming environment, which enables retrieval of de-identified individual-level microdata from DATASUS public repositories.^19^

As a nationwide administrative registry used for healthcare management and reimbursement, the SIA-SUS provides broad population coverage and has been used in epidemiological analyses of outpatient care in Brazil,^20^ consistent with the broader use of routinely collected health data in population-based research. However, such data are subject to known limitations, including variability in completeness, reporting delays, and limited clinical granularity. Within this framework, SIA-SUS presents operational characteristics that are important for interpretation. These include potential administrative latency in data entry, as information is recorded and consolidated through routine reporting processes; limited clinical granularity, as data are primarily collected for reimbursement purposes; and possible sensitivity to system-level disruptions, including underreporting during extreme events such as major floods.

The study population comprised all approved dispensing records — including the total quantity of units dispensed — for codeine, methadone, or morphine, extracted from the outpatient production microdata of the SIA/SUS for the state of Rio Grande do Sul, between January 2015 and December 2025. The study period comprised 132 consecutive calendar months. No outpatient opioid dispensing records were available for April 2021 due to a data availability issue in the SIA-SUS database. This time point was retained in the chronological sequence of the time series — preserving the monthly time index — but its outcome value was recorded as missing (NA) rather than entered as an observed zero. Consequently, the ecological time-series model was estimated using the 131 months with non-missing observations, as R's default listwise deletion (na.omit) automatically excluded this time point from model fitting while leaving the time index and seasonal structure of the remaining months unaffected. This approach aligns with established best practices for handling administrative data quality issues in ecological time-series studies.^21^^,^^22^ Data on the total number of outpatient opioid dispensations were extracted and subsequently aggregated by calendar month to construct the time series. No individual identifiers were retained in the analytical dataset, ensuring complete data anonymization in accordance with Brazilian data protection regulations.

### Outcome measures

The primary outcome was the monthly rate of outpatient opioid units dispensed per 10,000 inhabitants in Rio Grande do Sul. Monthly dispensing counts were calculated for all opioids combined and stratified by active pharmaceutical ingredients (codeine, methadone, and morphine). Rates were standardized using mid-year population estimates for Rio Grande do Sul obtained from the Brazilian Institute of Geography and Statistics (Instituto Brasileiro de Geografia e Estatística, IBGE) demographic projections for the corresponding years.

Secondary descriptive analyses characterized dispensing patterns by: (1) age group (categorized as ≤19 years, 20–39 years, 40–59 years, 60–79 years, ≥80 years) and (2) sex (male, female).

### Statistical analysis

#### Descriptive analysis

Descriptive statistics were first computed to characterize opioid dispensing patterns across the study period. Annual quantities of units dispensed volumes, and rates per 10,000 inhabitants were calculated for each opioid agent. Distributions by year, patient age, sex, and pharmaceutical formulation were summarized using frequencies and proportions.

#### Non-parametric retrospective ecological time-series analysis

A non-parametric estimation approach was used as the primary analytical method to evaluate changes associated with the flood-related disruption. The flood period (May 2024) was defined as the index event in the time series, corresponding to the month during which health services and infrastructure were most severely affected.

The models estimated:

1. The underlying secular trend in dispensing rates, modelled as a quadratic function of time to allow for non-linear changes over the study period;
2. The immediate level change associated with the flood month; and
3. The effect of each subsequent post-flood month individually, estimated through a series of monthly indicator variables rather than a single post-intervention trend term, allowing the trajectory of dispensing rates following the flood to be characterized flexibly across a 12-month post-event window, without imposing a pre-specified functional form on the pace or shape of recovery.

The coefficients were estimated to use the ordinary least squares method. The “lm()” function from the R base stats package was used, which fits the model by minimizing the sum of squared residuals between the observed and predicted values, according to the following formula:

$$Y_{t}=\beta_{0}+\beta_{1}.tempo_{t}+\beta_{2}.tempo_{t}^{2}+\sum_{h=0}^{12}\gamma_{h}.pos_{h,t}+\sum_{k=2}^{12}\delta_{k}.mes_{k,t}+\varepsilon_{t}$$

Where Y_t_ represents the outcome value in month t; β0 corresponds to the intercept; β1 and β2 capture the underlying secular trend, specified as a quadratic function of time; γ_h_ (h = 1, …, 12) represents the month-specific deviation in dispensing rates associated with the flood month (h = 0) and each of the 12 subsequent months (h = 1, …, 12), allowing the post-flood trajectory to be estimated non-parametrically rather than assuming a constant intervention effect; t; δ_k_ are the coefficients associated with each calendar month relative to the reference month, included to control for seasonality; and ε_t_ corresponds to the random error term. The time variable (“tempo”) was defined as the sequential number of months elapsed since the beginning of the series (1–132), with all months retained in the temporal structure regardless of data availability. The model was therefore fitted on 131 non-missing observations within a time index of 132 months. To control seasonality, the months of the year were included in the model as a categorical variable, using the “factor” function.

The presence of autocorrelation in the residuals was evaluated using the Durbin–Watson test, implemented by the dwtest() function from the lmtest” package. To correct for autocorrelation and heteroscedasticity, robust Newey–West standard errors were used. The number of lags was defined by the Newey–West rule (1994): floor [4 × (n/100)ˆ(2/9)],^23^ resulting in a lag of 4 for n = 131 observations, applied to the Newey–West estimator (1987) using the function of the same name from the ‘sandwich’ package. The p-values and confidence intervals for all coefficients, including the flood-related terms, were obtained from these robust Newey–West standard errors.

Regression coefficients (β) and their 95% confidence intervals were calculated to assess the magnitude and statistical significance of level and trend changes. Model fit was assessed using adjusted R2. The same model was applied to individual opioid agents and age groups (≤19, 20–39, 40–59, 60–79, ≥80 years) to assess heterogeneity in responses across medications and demographic strata.

All data processing and analyses were performed using R (version 4.5.0). Statistical significance was assessed at a two-sided α level of 0.05.

#### Control analysis

To contextualise whether the changes in opioid dispensing observed in Rio Grande do Sul coincided with broader contemporaneous temporal patterns, a placebo comparison state was selected using a multidimensional state-level similarity assessment conducted independently of post-flood opioid dispensing patterns. All Brazilian states were compared with Rio Grande do Sul using nine pre-event or structural indicators covering four domains: demographic characteristics (population size and age structure), socioeconomic characteristics (Human Development Index, gross domestic product, household income per capita, and literacy), territorial characteristics (population density and urbanisation), and health-system characteristics (number of SUS outpatient facilities per 100,000 inhabitants). Indicator-specific distances from Rio Grande do Sul were standardised and aggregated into domain-specific scores, which were then combined with equal domain weights to obtain an overall similarity score. States geographically close to Rio Grande do Sul and potentially exposed to shared regional meteorological conditions or indirect effects of the 2024 floods were considered unsuitable for the placebo comparison. Robustness of the comparator selection was assessed using an alternative specification assigning equal weight to each of the nine individual indicators. Post-flood opioid dispensing trajectories were not used in the comparator-selection procedure.

Minas Gerais was identified as the most structurally similar geographically eligible state and was therefore used for the placebo analysis. The same non-parametric model specification applied to Rio Grande do Sul—including the quadratic secular trend, calendar-month indicators for seasonality, and month-specific flood-related terms—was fitted to the Minas Gerais series at the corresponding calendar time points. This analysis assessed whether changes coinciding with May 2024 and the subsequent 12-month period were also observed in a state without direct exposure to the floods. Data handling followed the same procedures used for the primary series, including treatment of April 2021 as missing rather than as an observed zero; the model was therefore fitted to 131 non-missing monthly observations within the 132-month time index.

#### Sensitivity analysis

##### COVID-19 period exclusion

To evaluate the robustness of the findings and assess the potential influence of the COVID-19 pandemic on the estimation of secular trends, a sensitivity analysis was conducted by excluding observations from January 2020 through December 2021. The ecological time-series models were then re-estimated using the same specification as the primary analysis, including the underlying quadratic secular trend, monthly indicator variables to account for seasonality, and the full set of flood-related shock terms estimating the effect of each post-flood month individually. Following the exclusion of the pandemic period, the time index was recalculated to preserve the temporal ordering of the remaining observations. To account for potential heteroskedasticity and serial autocorrelation, standard errors were estimated using the Newey–West heteroskedasticity- and autocorrelation-consistent estimator,^24^ with the lag length selected automatically according to the rule proposed by Newey and West.

##### Linear vs. quadratic trend

To assess whether the non-linear (quadratic) secular trend used in the primary analysis was necessary to adequately capture the underlying dispensing pattern, or whether a linear trend would provide comparable fit, a sensitivity analysis was performed comparing quadratic and linear trend models for each outcome. The linear specification retained the same structure as the primary model — monthly seasonality indicators and the full set of flood-related shock terms — omitting only the squared time term. As in the primary analysis, April 2021 was treated as missing (NA) and excluded from model estimation for both specifications, which were fitted to the identical set of non-missing observations for each outcome.

Model comparison was based on the Akaike Information Criterion (AIC), the Bayesian Information Criterion (BIC), and the adjusted R2 for each specification. The difference in AIC and BIC between models (ΔAIC and ΔBIC, linear minus quadratic) determined which specification better balanced fit against complexity; positive values indicated the quadratic model was preferred. The quadratic trend was considered preferable to the linear specification only when ΔAIC and ΔBIC agreed in direction and both exceeded a magnitude of 2, corresponding to positive evidence on the Burnham and Anderson and Raftery scales, respectively. Otherwise, the comparison was classified as inconclusive, and the linear specification could not be ruled out as an equally parsimonious alternative.

### Ethical considerations

This study used publicly available, anonymized secondary data and did not involve direct contact with human participants. According to Brazilian regulations for research using secondary administrative data, formal ethics committee approval was not required.

### Role of the funding source

The funder had no role in the design of the study; the collection, analysis, or interpretation of the data; the writing of the manuscript; or the decision to submit the manuscript for publication.

## Results

### Overall opioid dispensing trends

Between January 2015 and December 2025, a total of 6,572,838 (100.0%) units outpatient opioid dispensations were recorded in the SIA-SUS of Rio Grande do Sul. Annual dispensing volumes demonstrated a sustained increase throughout the study period, rising from 623,222 (9.5%) dispensed units in 2020 to 894,075 (13.6%) in 2024, corresponding to a 43.46% overall increase. Compared with 2023 (12.0%; n = 792,029), dispensed units in 2024 increased by 12.88%.

Overall, codeine accounted for 52.0% of all dispensed units (n = 3,416,000), followed by morphine (27.8%, n = 1,827,609) and methadone (20.2%, n = 1,329,229). Dispensed units were most frequent among individuals aged 40–59 years (52.4%, n = 3,441,242), followed by those aged 60–79 years (33.6%, n = 2,208,373). Female accounted for 63.6% (n = 4,179,180) of all dispensed units. Detailed descriptive characteristics of outpatient opioid dispensed units, stratified by opioid agent, are summarized in Table 1.

**Table 1:** Descriptive characteristics of units outpatient opioid dispensations in Rio Grande do Sul, Brazil, January 2015–December 2025.

Variable	Total–n (%)	Codeine–n (%)	Morphine–n (%)	Methadone–n (%)
Total dispensation	6,572,838 (100.0)	3,416,000 (52.0)	1,827,609 (27.8)	1,329,229 (20.2)
Year				
2015	197,079 (3.0)	60,438 (1.8)	96,200 (5.3)	40,441 (3.0)
2016	276,198 (4.2)	119,765 (3.5)	106,723 (5.8)	49,710 (3.7)
2017	386,539 (5.9)	197,358 (5.8)	122,208 (6.7)	66,973 (5.0)
2018	404,518 (6.2)	190,550 (5.6)	135,794 (7.4)	78,174 (5.9)
2019	478,437 (7.3)	240,182 (7.0)	154,667 (8.5)	83,588 (6.3)
2020	623,222 (9.5)	317,331 (9.3)	167,822 (9.2)	138,069 (10.4)
2021	684,550 (10.4)	349,550 (10.2)	182,764 (10.0)	152,236 (11.5)
2022	723,338 (11.0)	386,882 (11.3)	178,388 (9.8)	158,068 (11.9)
2023	792,029 (12.0)	426,795 (12.5)	203,747 (11.1)	161,487 (12.2)
2024	894,075 (13.6)	495,872 (14.5)	212,905 (11.6)	185,298 (13.9)
2025	1,112,853 (16.9)	631,277 (18.5)	266,391 (14.6)	215,185 (16.2)
Age group (years)				
0–19	23,987 (0.4)	7704 (0.2)	7997 (0.4)	8286 (0.6)
20–39	666,915 (10.1)	225,454 (6.6)	238,871 (13.1)	202,590 (15.2)
40–59	3,441,242 (52.4)	1,714,176 (50.2)	1,004,811 (55.0)	722,255 (54.3)
60–79	2,208,373 (33.6)	1,321,678 (38.7)	513,794 (28.1)	372,901 (28.1)
80+	232,321 (3.5)	146,988 (4.3)	62,136 (3.4)	23,197 (1.8)
Sex				
Female	4,179,180 (63.6)	2,240,025 (65.6)	1,185,784 (64.9)	753,371 (56.7)
Male	2,393,658 (36.4)	1,175,975 (34.4)	641,825 (35.1)	575,858 (43.3)

Note: Data are presented as absolute counts (n) and column percentages (%). For this descriptive analysis, percentages represent the proportion of dispensations within each category (Year, Age Group, or Sex) relative to the total for that specific opioid agent (column total).

### Non-parametric ecological time-series analysis of units opioid dispensing during the 2024 floods in Rio Grande do sul

The non-parametric time-series model indicated that the 2024 flooding was associated with a marked, though transient disruption in outpatient opioid dispensing (Fig. 1, Table 2). Dispensing collapsed almost entirely in the month of the flood (May 2024), recovered to approximately 78.5% of the initial shock by the following month, and then largely normalized, with a further significant rebound roughly five months after the flood. At the time of the flood impact (May 2024), dispensing fell sharply with an estimated reduction of −68.86 dispensed units per 10,000 population relative to the projected counterfactual trend (95% CI: −73.57 to −64.15; p < 0.001). A near-total collapse in the system’s dispensing capacity this disruption attenuated rapidly: by June 2024, the deficit had narrowed to −14.81 (95% CI: −21.12 to −8.51; p < 0.001), corresponding to a recovery of approximately 78.5% of the initial shock.

**Fig. 1:**
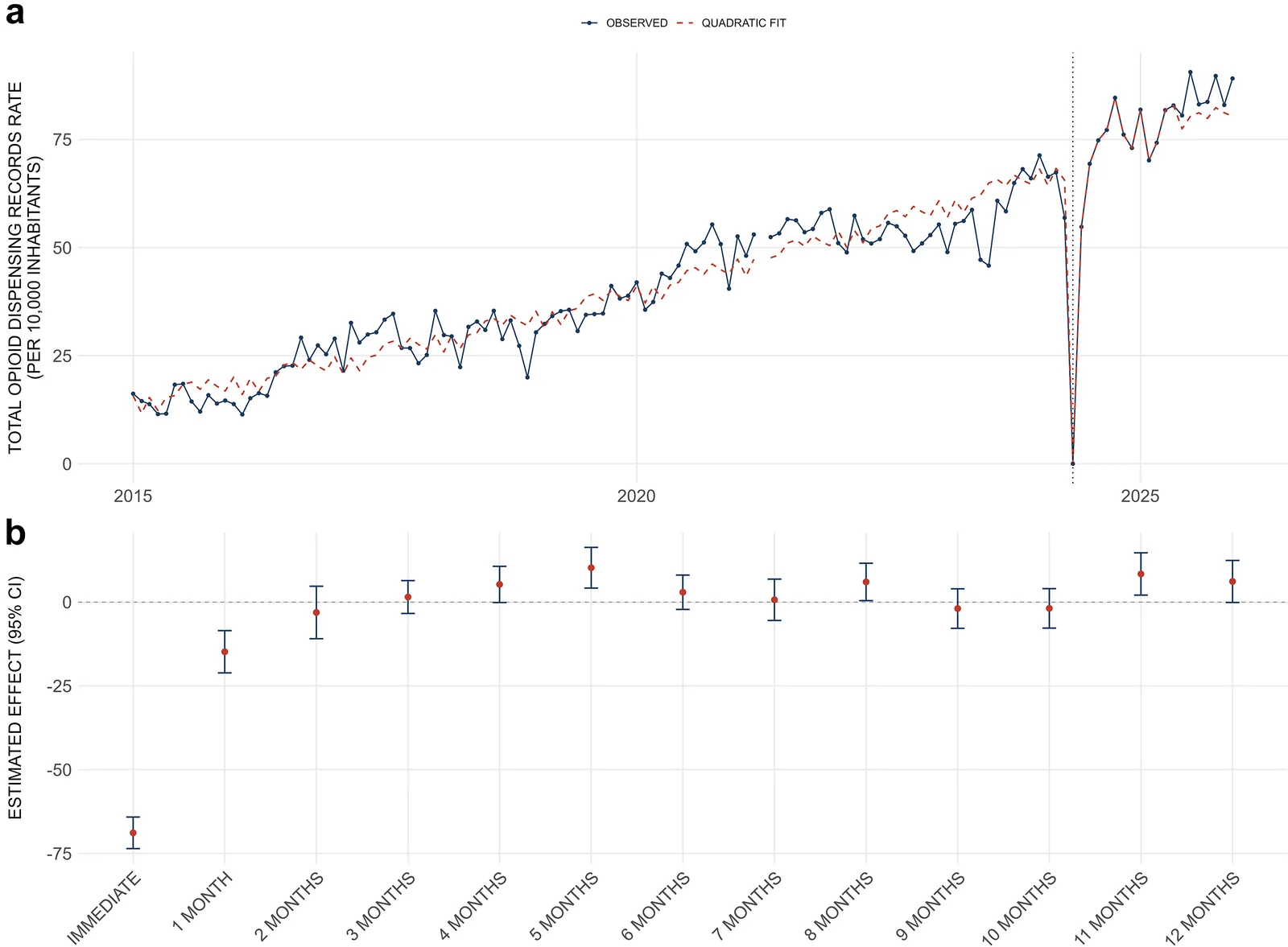
Observed and modelled total opioid dispensing rates and estimated monthly effects following the 2024 floods in Rio Grande do Sul. Note: (a) Observed monthly total opioid dispensing rate (per 10,000 inhabitants) with quadratic trend fit, 2015–2025; the vertical dotted line indicates the flood event (May 2024). (b) Estimated monthly effects on dispensing rate (95% CI) relative to the counterfactual trend, from immediate to 12 months post-flood.

**Table 2:** Non-parametric ecological time-series analysis of outpatient opioid dispensing after the May 2024 flooding, Rio Grande do Sul, Brazil.

Variable	Flood month effect (May 2024) in dispensing rates (95% CI)	One-month post-flood effect (June 2024) in dispensing rates (95% CI)	Two-month post-flood effect (July 2024) in dispensing rates (95% CI)	Three-month post-flood effect (August 2024) in dispensing rates (95% CI)	Four-month post-flood effect (September 2024) in dispensing rates (95% CI)	Five-month post-flood effect (October 2024) in dispensing rates (95% CI)	Six-month post-flood effect (November 2024) in dispensing rates (95% CI)	Seven-month post-flood effect (December 2024) in dispensing rates (95% CI)	Eight-month post-flood effect (January 2025) in dispensing rates (95% CI)	Nine-month post-flood effect (February 2025) in dispensing rates (95% CI)	Ten-month post-flood effect (March 2025) in dispensing rates (95% CI)	Eleven-month post-flood effect (April 2025) in dispensing rates (95% CI)	Twelve-month post-flood effect (May 2025) in dispensing rates (95% CI)	Adjusted R^2^	Durbin–Watson test
Total opioids	−68.8616 (−73.5745 to −64.1486)^c^	−14.8123 (−21.1165 to −8.5081)^c^	−3.0815 (−10.9156 to 4.7526)	1.5276 (−3.3752 to 6.4304)	5.2929 (−0.1105 to 10.6963)	10.2716 (4.1919–16.3512)^b^	2.9564 (−2.1637 to 8.0764)	0.6997 (−5.4703 to 6.8698)	6.0301 (0.4488–11.6115)^a^	−1.9168 (−7.8104 to 3.9769)	−1.8599 (−7.7486 to 4.0288)	8.4096 (2.0984–14.7207)^b^	6.1760 (−0.0968 to 12.4487)	0.936	0.758
By opioid agent															
Codeine	−37.6558 (−40.5807 to −34.7310)^c^	−2.2904 (−5.3630 to 0.7823)	−0.2611 (−4.2695 to 3.7473)	1.5079 (−1.2681 to 4.2838)	2.2208 (−1.4600 to 5.9016)	4.9629 (0.7078–9.2180)^a^	−0.5930 (−3.6391 to 2.4532)	−2.0688 (−5.6824 to 1.5448)	5.2659 (2.0110–8.5208)^b^	0.8190 (−2.7954 to 4.4334)	2.2368 (−1.3965 to 5.8701)	5.9152 (2.0518–9.7787)^b^	3.4669 (−0.4961 to 7.4299)	0.948	0.677
Morphine	−16.5745 (−17.7999 to −15.3492)^c^	−7.4682 (−9.0963 to −5.8400)^c^	−2.9230 (−4.6720 to −1.1740)^b^	−2.6133 (−4.0037 to −1.2228)^c^	0.4213 (−1.2250 to 2.0676)	1.3409 (0.0863–2.5955)^a^	1.7952 (0.3465–3.2439)^a^	3.7884 (1.9963–5.5806)^c^	1.7498 (0.0783–3.4213)^a^	1.6026 (0.0559–3.1493)^a^	−1.5175 (−3.1784 to 0.1434)	1.0307 (−0.6997 to 2.7612)	1.0457 (−0.5007 to 2.5921)	0.831	1.286
Methadone	−14.6312 (−15.8816 to −13.3807)^c^	−5.0538 (−7.2382 to −2.8694)^c^	0.1026 (−2.6278 to 2.8330)	2.6330 (0.8146–4.4514)^b^	2.6508 (1.5552–3.7463)^c^	3.9678 (2.6189–5.3168)^c^	1.7541 (0.1857–3.3225)^a^	−1.0199 (−2.4169 to 0.3771)	−0.9855 (−2.3572 to 0.3861)	−4.3384 (−5.9078 to −2.7690)^c^	−2.5792 (−4.0734 to −1.0849)^c^	1.4636 (−0.3823 to 3.3095)	1.6634 (0.0313–3.2954)^a^	0.822	0.699
By age group															
0–19 years	−0.1895 (−0.2989 to −0.0802)^c^	−0.0656 (−0.1478 to 0.0167)	0.1101 (0.0305–0.1896)^b^	−0.0945 (−0.1973 to 0.0083)	−0.0887 (−0.1779 to 0.0004)	−0.0680 (−0.1562 to 0.0203)	−0.0519 (−0.1525 to 0.0486)	−0.0044 (−0.0855 to 0.0767)	−0.0335 (−0.1181 to 0.0512)	−0.1622 (−0.2820 to −0.0425)^b^	−0.0165 (−0.1207 to 0.0876)	0.0975 (−0.0238 to 0.2188)	−0.0250 (−0.1395 to 0.0896)	0.079	0.731
20–39 years	−6.4240 (−7.0960 to −5.7519)^c^	−2.3859 (−3.1601 to −1.6117)^c^	−1.2218 (−2.2208 to −0.2228)^a^	0.6912 (−0.0062 to 1.3886)	0.9258 (0.2528–1.5988)^b^	1.3910 (0.6517–2.1302)^c^	−0.1015 (−0.8129 to 0.6099)	0.0498 (−0.7031 to 0.8026)	0.5263 (−0.2380 to 1.2906)	−0.3061 (−1.1648 to 0.5527)	−0.1212 (−0.9597 to 0.7172)	0.1958 (−0.7949 to 1.1866)	0.4191 (−0.4419 to 1.2800)	0.808	1.033
40–59 years	−36.1749 (−38.6219 to −33.7279)^c^	−7.9578 (−11.3228 to −4.5928)^c^	−1.0902 (−4.9913 to 2.8109)	1.7428 (−0.9068 to 4.3925)	1.8993 (−1.1048 to 4.9034)	5.3809 (2.0302–8.7316)^b^	0.6999 (−2.0153 to 3.4150)	−0.3472 (−3.6701 to 2.9757)	2.9410 (0.0575–5.8245)^a^	−1.6173 (−4.5811 to 1.3464)	−1.0102 (−4.1252 to 2.1049)	2.8516 (−0.6856 to 6.3888)	2.0247 (−1.2814 to 5.3309)	0.933	0.787
60–79 years	−23.7092 (−25.9468 to −21.4716)^c^	−4.3495 (−6.9472 to −1.7519)^b^	−0.6641 (−3.8762 to 2.5480)	−0.3779 (−2.5417 to 1.7860)	2.1973 (−0.0212 to 4.4158)	3.7374 (1.2351–6.2397)^b^	2.7389 (0.4790–4.9989)^a^	0.7775 (−1.7771 to 3.3321)	2.8554 (0.4966–5.2141)^a^	0.1552 (−2.3687 to 2.6792)	−0.2524 (−2.7874 to 2.2826)	4.7982 (2.1883–7.4081)^c^	3.6403 (0.7817–6.4989)^a^	0.923	0.656
80+ years	−2.3640 (−2.8077 to −1.9203)^c^	−0.0535 (−0.3983 to 0.2913)	−0.2154 (−0.5189 to 0.0882)	−0.4341 (−0.8362 to −0.0320)^a^	0.3592 (−0.0728 to 0.7913)	−0.1697 (−0.6333 to 0.2939)	−0.3290 (−0.7311 to 0.0731)	0.2240 (−0.2951 to 0.7431)	−0.2591 (−0.7076 to 0.1894)	0.0136 (−0.4512 to 0.4784)	−0.4595 (−0.9684 to 0.0493)	0.4664 (−0.0420 to 0.9749)	0.1169 (−0.4434 to 0.6771)	0.755	0.920

a p < 0.05.

b p < 0.01.

c p < 0.001.

From July through September 2024, the estimated effects were not statistically significant, ranging from −3.08 (95% CI: −10.92 to 4.75) in July to +5.29 (95% CI: −0.11 to 10.70) in September, indicating that dispensing had returned to levels consistent with the counterfactual trend, a period of relative stabilization following the initial disruption.

A significant rebound was subsequently observed in October 2024, approximately five months after flood, with dispensing rates 10.27 dispensed units per 10,000 higher than expected under the counterfactual trend (95% CI: 4.19 to 16.35; p < 0.01). This was followed by non-significant fluctuations in November and December 2024 (+2.96 and + 0.70, respectively), and then another significant increase in January 2025 (+6.03; 95% CI: 0.45 to 11.61; p < 0.05). Effects in February and March 2025 were again non-significant (−1.92 and −1.86, respectively), while April 2025 showed a significant increase of 8.41 (95% CI: 2.10 to 14.72; p < 0.01). By May 2025 — twelve months after the flood — the estimated effect was no longer statistically significant, showing a +6.18 dispensed units per 10,000 population increase relative to the counterfactual trend (95% CI: −0.10 to 12.45).

Model diagnostics indicated excellent fit, with an adjusted R2 = 0.936, indicating that 93.6% of the variance in total opioid dispensing rates was explained by the model. However, there remained strong evidence of positive residual autocorrelation, as indicated by the Durbin–Watson test (DW = 0.758, p < 0.001), reinforcing the use of Newey–West robust standard errors to correct the effect estimates and confidence intervals reported above.

### Opioid-specific disruption patterns

#### By active pharmaceutical ingredients

When stratified by active pharmaceutical ingredients, distinct disruption-and-recovery patterns were observed across the three opioid agents, with codeine recovering most rapidly, methadone recovering within two months, and morphine showing a more gradual prolonged decline, reflecting substantial differences in magnitude, duration and trajectory of the estimated effects (Fig. 2, Table 2).

**Fig. 2:**
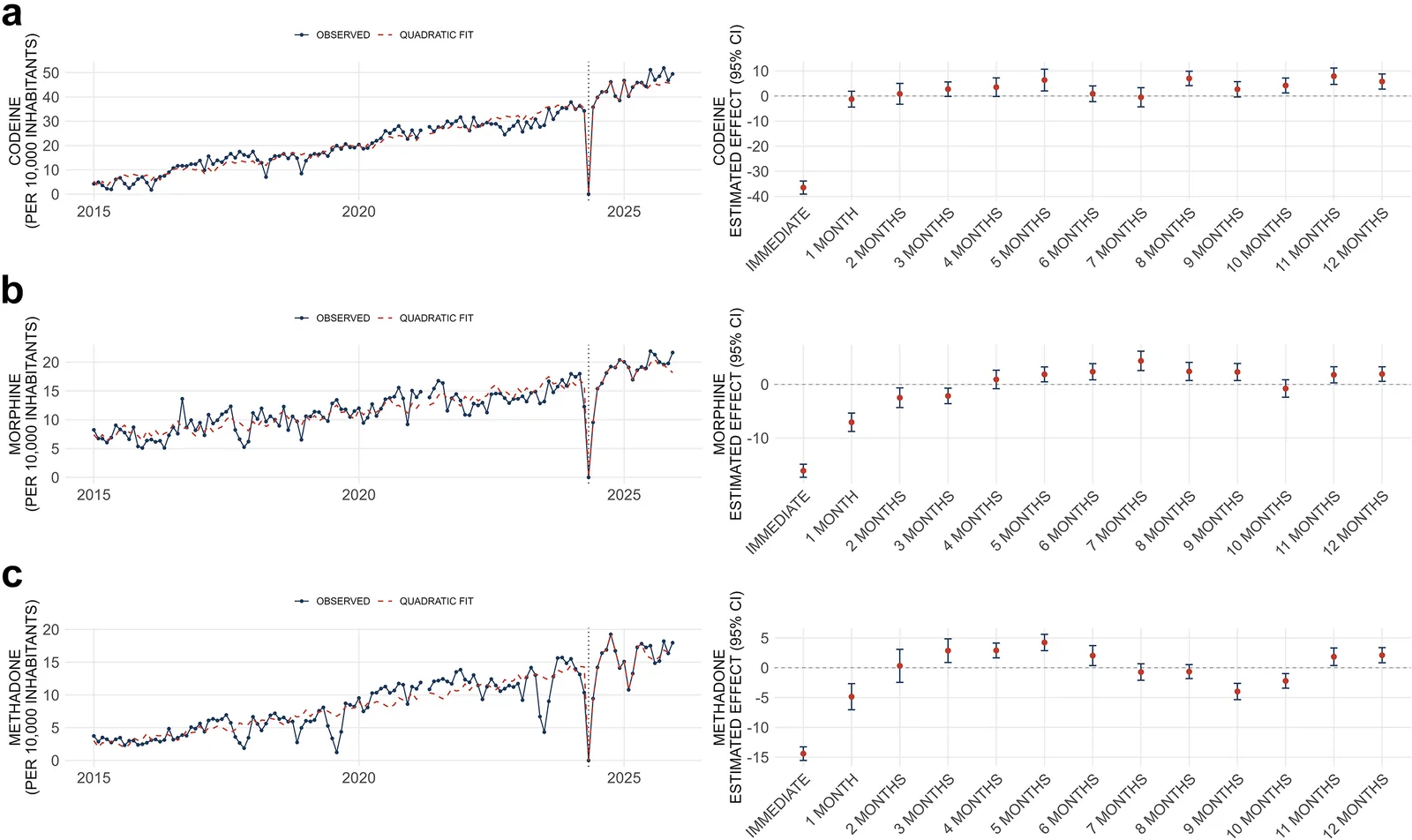
Observed and modelled total opioid dispensing rates and estimated monthly effects following the 2024 floods, by active pharmaceutical ingredients in Rio Grande do Sul. Note: Monthly dispensing rate (per 10,000 inhabitants) with quadratic trend fit for (a) codeine, (b) morphine, and (c) methadone. The vertical dotted line indicates the 2024 flood event (May 2024), and estimated monthly effects on dispensing rate (95% CI) relative to the counterfactual trend, from immediate to 12 months post-flood.

A significant immediate reduction in dispensing of codeine was observed in May 2024, with an estimated effect of −37.66 dispensed units per 10,000 population relative to the counterfactual trend (95% CI: −40.58 to −34.73; p < 0.001). This deficit narrowed sharply by June 2024 corresponding a recovery of approximately 93.3% of the initial shock, and the effect was no longer statistically significant from June through September 2024, with estimates ranging from −2.29 to +2.22. A significant rebound occurred in October 2024 (+4.96; 95% CI: 0.71 to 9.22; p < 0.05), followed by non-significant effects in November and December 2024 (−0.59 and −2.07, respectively). A second significant increase was observed in January 2025 (+5.27; 95% CI: 2.01 to 8.52; p < 0.01), after which effects in February and March 2025 were non-significant (+0.82 and + 2.24, respectively). April 2025 showed a further significant increase of +5.92 (95% CI: 2.05 to 9.78; p < 0.01), while by May 2025 the estimated effect had returned to non-significance (+3.47; 95% CI: −0.50 to 7.43).

Morphine dispensing showed a significant immediate reduction in May 2024 (−16.57; 95% CI: −17.80 to −15.35; p < 0.001), which recovered more gradually than the other agents: the deficit no he deficit narrowed to −7.47 in June (∼54.9% recovery), −2.92 in July (∼82.4% recovery), and −2.61 in August (∼84.2% recovery), with effects remaining significantly negative throughout this period (all p < 0.01 or lower). The effect became non-significant in September 2024 (+0.42; 95% CI: −1.23 to 2.07), before turning significantly positive from October through January 2025, with increases ranging from +1.34 to +3.79 (all p < 0.05). Effects remained significantly positive in February 2025 (+1.60; p < 0.05) but became non-significant thereafter, with estimates of −1.52 in March, +1.03 in April, and +1.05 in May 2025, none reaching statistical significance.

Methadone dispensing exhibited a significant immediate reduction in May 2024 (−14.63; 95% CI: −15.88 to −13.38; p < 0.001), which had already recovered to approximately 65.5% of the initial shock by June 2024 (−5.05; p < 0.001) before returning to the counterfactual trend in July 2024 (+0.10). A significant rebound followed from August through October 2024, with increases of +2.63 (p < 0.01), +2.65 (p < 0.001), and +3.97 (p < 0.001), respectively, and a further significant increase in November 2024 (+1.75; p < 0.05). Effects were non-significant in December and January 2025 (−1.02 and −0.99, respectively), followed by a significant reversal — a decline — in February and March 2025 (−4.34 and −2.58, respectively; both p < 0.001). Effects returned to non-significance in April 2025 (+1.46), before a significant increase reemerged in May 2025 (+1.66; 95% CI: 0.03 to 3.30; p < 0.05).

Model fit was excellent across all three agents, with adjusted R2 values of 0.948 (codeine), 0.831 (morphine), and 0.822 (methadone). Durbin–Watson statistics indicated residual autocorrelation in all models (codeine: DW = 0.677; morphine: DW = 1.286; methadone: DW = 0.699), reinforcing the appropriateness of the robust, non-parametric approach used to estimate the confidence intervals reported above.

#### By age groups

Nonparametric analyses stratified by age group revealed significant declines (p < 0.001) during the month of the floods across all age groups evaluated, with the magnitude of the decline increasing with age—ranging from −0.19 (0–19 years) to −36.17 (40–59 years). The pace and completeness of subsequent recovery, however, differed considerably among the age groups (Table 2).

One month after the disaster, recovery remained partial in the three largest magnitude groups: the 20–39 age group had recovered approximately 62.9% of its initial deficit (−2.39; still significant, p < 0.001), the 40–59 age group approximately 78.0% (−7.96; p < 0.001), and the 60–79 age group approximately 81.7% (−4.35; p < 0.01), indicating that the initial impact on dispensing persisted, in attenuated form, in these groups. In the youngest (0–19 years) and oldest (80+ years) age groups, the effect was no longer significant starting in the second month.

Despite this relatively fast initial rebound, the trajectories diverged over the following month. In the 20–39 and 40–59 age groups, the coefficients returned to positive values as early as the third month after the flood (+0.69 and + 1.74, respectively), although these were not statistically significant, and they stabilized around zero in the following months—except for a new significant increase in October 2024 for the 20–39 age group (+1.39; p < 0.001) and in January 2025 for the 40–59 age group (+2.94; p < 0.05).

In contrast, the 60–79 age group, despite recovering the largest share of its initial deficit by month one, showed the most sustained recovery pattern: the coefficient turned positive starting in the fourth month (+2.20, not significant), but began to show statistically significant increases in October (+3.74; p < 0.01), November (+2.74; p < 0.05), and January 2025 (+2.86; p < 0.05), culminating in the largest increase in the series in the eleventh month after the flood, April 2025 (+4.80; p < 0.001), with another significant increase in May 2025 (+3.64; p < 0.05). This pattern suggests a delayed rebound effect in medication dispensing for this age group, possibly associated with the resumption of chronic treatments that had been put on hold during the acute phase of the disaster.

In the 0–19 age group, the pattern was qualitatively different: a slight but significant increase was observed as early as the second month (+0.11; p < 0.01), followed by another significant decrease only in the ninth month (−0.16; p < 0.01), with no clear trend toward sustained recovery over the period.

Finally, the 80+ age group showed the most stable trend after the initial month, with only one additional significant decrease in the third month following the flood (−0.43; p < 0.05) and no other coefficients reaching statistical significance by the end of the 12-month period analysed. The models showed good overall fit, with an adjusted R2 ranging from 0.079 (ages 0–19) to 0.933 (ages 40–59), and the Durbin–Watson statistics (0.656–1.033) indicate the presence of positive residual autocorrelation in all age groups, which should be taken into account when interpreting the confidence interval.

### Control analysis

The control series for Minas Gerais is presented as a comparative benchmark to contextualize the findings of the primary analysis and to aid in distinguishing intervention-specific effects from broader temporal trends.

A total of 13,031,430 (100.0) opioid dispensations were recorded in the state of Minas Gerais between January 2015 and December 2025. Morphine was the most frequently dispensed opioid, representing 42.8% (n = 5,579,570), followed by codeine at 38.7% (n = 5,043,552) and methadone at 18.5% (n = 2,408,308). Regarding age groups, the highest proportion of dispensations was among individuals aged 40–59 years, 47.8% (n = 6,224,034), followed by the 60 to 79 age group at 36.4% (n = 4,748,524) and the 20 to 39 age group at 10.2% (n = 1,331,055). The female sex accounted for 56.1% (n = 7,313,422) of the number of opioid dispensations (Supplementary Table S1).

Inspection of the monthly series revealed a pronounced but temporary decline in recorded opioid dispensing in Minas Gerais in 2019, followed by a recovery in 2020. This state-specific fluctuation predated the 2024 floods and was considered likely related to disruptions in the state pharmaceutical supply chain rather than to the event evaluated in the primary analysis.

The control series for the state of Minas Gerais showed no statistically significant interruption in total opioid dispensing in any of the thirteen months evaluated, including the month corresponding to the flood event in Rio Grande do Sul (+0.39; 95% CI −10.50 to 11.27) and the entire subsequent twelve-month period. This pattern of no effect is consistent with the state’s role as a comparative benchmark, reinforcing that the changes observed in the intervention series reflect the specific impact of the disaster, rather than a broader temporal trend that would also have affected unaffected regions. The model fit for the total series, however, was quite limited (adjusted R2 = 0.040), with high residual autocorrelation (Durbin–Watson = 0.198), which calls for caution in interpreting the magnitude of the coefficients (Supplementary Fig. S1).

Among the opioid agents analysed individually, methadone stood out, showing statistically significant and persistent reductions at multiple points in the time series—two months (−3.76; p < 0.001), three months (−4.76; p < 0.001), eight months (−2.04; p < 0.05), ten months (−3.22; p < 0.01), and twelve months (−2.56; p < 0.05) after May 2024—despite the absence of any flooding events in that state. This finding suggests a downward trend in methadone dispensing during the period, possibly related to regulatory or market factors independent of the climate disaster, and should not be confused with an intervention effect. Codeine and morphine, on the other hand, did not show any statistically significant coefficients throughout the entire analysed period, with weak or negative model fit (adjusted R2 of −0.089 and 0.088, respectively) (Supplementary Fig. S2).

When stratified by age group, the 0–19 age group was the only one to show a pattern of recurring significant increases—in the index month (+0.20; p < 0.05), in the first month (+0.24; p < 0.001), in the second month (+0.15; p < 0.01), in the fifth month (+0.42; p < 0.001), in the sixth month (+0.24; p < 0.01), and in the eighth month (+0.21; p < 0.05)—indicating a trend of sustained growth in paediatric prescriptions that is unrelated to the disaster in Minas Gerais and contrasts with the pattern observed in the intervention series for this same age group. The other age groups (20–39, 40–59, 60–79, and 80+ years) mostly showed non-significant coefficients with no consistent direction, with isolated exceptions—such as the decrease in the 20–39 age group in the tenth month (−1.30; p < 0.05) and the increases in the 80+ age group in the third, fifth, sixth, and eighth months—suggesting random fluctuations rather than a systematic trend. The model adjustments for the age groups in the control series were, in general, modest (adjusted R2 ranging from −0.026 to 0.480), with Durbin–Watson statistics indicating residual autocorrelation in almost all groups, except for the 0–19 age group (DW = 1.494), whose model showed better specification (Supplementary Table S2).

### Sensitivity analysis

The sensitivity analysis, in which the years 2020–2021 were excluded to minimize potential confounding effects related to the COVID-19 pandemic, generally confirmed the robustness of the main findings. The significant reduction in total opioid dispensing during the month of the flood remained evident and was even more pronounced (−68.70; p < 0.001), with the effect persisting into the first month after the disaster (−13.97; p < 0.001), a pattern consistent with that observed in the main analysis. Unlike the original model, however, this sensitivity analysis revealed a statistically significant recovery occurring earlier, as early as the fourth month (+5.73; p < 0.001) and the fifth month (+10.52; p < 0.001), in addition to further significant reductions in the ninth and tenth months (−6.03 and −6.39, respectively; p < 0.01), suggesting a less linear recovery trajectory than that suggested by the model using the complete time series. The model fit improved substantially after excluding the pandemic period (adjusted R2 = 0.950, compared with 0.040 for the full time series), indicating that much of the unexplained variability in the original model was in fact associated with the atypical fluctuations of 2020–2021.

By opioid agent, the pattern of an initial decline followed by partial recovery remained consistent for codeine, morphine, and methadone, all of which showed highly significant reductions in the index month (p < 0.001) and high model fit (adjusted R2 between 0.836 and 0.954). Morphine stood out for having a more prolonged decline, with significant reductions persisting through the third month after the flood (−2.62; p < 0.001), while codeine and methadone no longer showed a significant effect starting in the second month, although methadone again showed significant reductions in months eight through ten (p < 0.05 to p < 0.001).

When stratified by age group, the results of the sensitivity analysis were largely consistent with those of the main analysis, both in terms of the direction and magnitude of the coefficients, reinforcing that the findings are not artifacts of the pandemic period included in the original data series. The 20–39, 40–59, and 60–79 age groups maintained significant reductions in the month of the flood and the following month, with gradual recovery beginning in the fourth or fifth month, and the 60–79 age group again exhibited the pattern of delayed and sustained recovery, with significant increases between the fifth and eleventh months post-disaster. Model fit for the age groups was also considerably higher than that of the complete series (adjusted R2 ranging from 0.096 to 0.954, except for the 0–19 age group), confirming that excluding the pandemic period significantly reduced statistical noise in the estimates, without substantially altering the conclusions regarding the flood’s impact on opioid dispensing (Supplementary Table S3).

A comparative analysis was conducted between the linear and quadratic models to identify which one provided the best fit to the data. The models were evaluated based on their performance indicators, and after the comparison, it was found that the quadratic model performed better than the linear model. Of the nine variables analysed, six showed a better fit with the quadratic model. Therefore, this model was selected (Supplementary Table S4).

## Discussion

In this population-based analysis, the May 2024 floods were associated with a marked and immediate decline in recorded outpatient opioid dispensing in Rio Grande do Sul, followed by early stabilization and an uneven recovery that varied across opioid agents. The immediate decline remained evident in analyses excluding the COVID-19 period and was not accompanied by a comparable contemporaneous reduction in Minas Gerais, providing contextual support for a regionally specific disruption. Although Minas Gerais exhibited a marked temporary decline in recorded opioid dispensing in 2019, this fluctuation predated the study interruption and was considered likely related to a state-level disruption in the pharmaceutical supply chain, rather than to the 2024 floods. To our knowledge, comparable studies examining outpatient opioid dispensing after climate-related disasters in universal public health systems in middle-income settings are lacking. These findings therefore extend the limited evidence on medication access during disasters and highlight the vulnerability of controlled-medicine pathways and treatment continuity during large-scale climate-related emergencies.

The observed decline likely reflects disruptions in both health-care delivery and administrative reporting. Flood and storm disasters can damage health facilities, interrupt pharmaceutical supply chains, restrict population mobility, displace patients, and reduce workforce availability.^8^^,^^9^^,^^25^^,^^26^ In Rio Grande do Sul, official assessments documented hundreds of affected health-care facilities and extensive road closures during the acute phase of the floods.^27^ These conditions are likely to have substantially reduced actual dispensing activity, particularly in the most severely affected settings. At the same time, because SIA-SUS records outpatient procedures through monthly administrative reporting cycles, the floods may also have impaired the capacity of facilities to enter, process, and submit data, further reducing recorded activity.^19^, ^20^, ^21^^,^^28^ Taken together, the magnitude of the decline and the documented disruption are consistent with a major system-level shock affecting both dispensing activity and administrative recording.

Following the marked reductions in May and June 2024, dispensing returned to levels that did not differ significantly from the counterfactual between July and September, followed by an uneven trajectory with intermittent above-expected increases. This pattern is consistent with the progressive restoration of health-care and pharmaceutical services after the acute disruption, potentially involving the resumption of interrupted treatments and the fulfilment of delayed health-care needs.^9^, ^10^, ^11^, ^12^^,^^26^ Some subsequent increases may also have reflected delayed administrative processing, reporting regularization, or the submission of procedures that had not been recorded during the most critical phase of the floods.^19^, ^20^, ^21^^,^^28^ Overall, the trajectory suggests progressive but uneven restoration of dispensing activity rather than a rapid or uniform return to normal operation.

The magnitude and duration of the disruption varied across opioid agents. Codeine, which accounted for most dispensing events, showed an acute decline followed by a comparatively rapid return towards expected levels. Morphine exhibited the most prolonged initial disruption, with below-expected dispensing extending beyond the flood month, whereas methadone estimates were more variable and less precise. Intermittent above-expected values during the subsequent period were not consistent across agents and should not be interpreted as evidence of a uniform post-flood increase. This heterogeneity may reflect agent-specific differences in baseline dispensing volumes, clinical indications, service organisation, and the pathways through which each medication is provided.

The timing of the acute disruption was broadly similar across age groups, although the magnitude and precision of the estimates varied. The largest absolute reductions were observed among adults aged 40–59 years and 60–79 years, who also accounted for a substantial proportion of dispensing activity. Higher baseline dispensing volumes may therefore partly explain the larger absolute changes in these groups. The contemporaneous decline across all age groups in Rio Grande do Sul is consistent with a system-wide acute disruption rather than an effect confined to a specific demographic subgroup. However, age-specific post-flood trajectories should be interpreted cautiously. In the comparison state, the 0–19-year age group showed recurring increases over the same period, including during the index month, indicating that some age-specific temporal changes may reflect background dispensing trends unrelated to the disaster.

Viewed within the broader disaster-health literature, these findings suggest that the effects of climate-related emergencies can extend to routine processes required for the management of chronic and complex conditions. Previous studies have documented disruptions in health-care delivery, pharmaceutical services, and continuity of care following floods, storms, and other natural disasters.^8^, ^9^, ^10^, ^11^, ^12^^,^^25^^,^^26^ Our study adds evidence on outpatient opioid dispensing within a universal public health system in a middle-income setting. Brazil’s Unified Health System provides a distinctive real-world setting in which changes in system-wide dispensing activity can be examined across a large and heterogeneous population.^15^^,^^16^ This setting may be particularly informative for other low- and middle-income countries, where comparable population-level evidence is often limited and the effects of climate-related disasters on medication delivery may be difficult to estimate.

Because opioids are subject to strict prescribing and dispensing controls, their dispensing may also function as a tracer of the resilience of medication-delivery systems during acute disruptions.^29^ Although opioids cannot be considered a direct proxy for all medicines, disruption in their delivery may reveal vulnerabilities relevant to other essential treatments that depend on functioning supply, authorisation, information, and dispensing pathways. The abrupt decline followed by an uneven restoration therefore highlights the susceptibility of medication-delivery systems to simultaneous disruptions in infrastructure, workforce, transportation, pharmaceutical supply chains, and health-information systems. Although context-specific, the experience of Rio Grande do Sul may provide an informative case for regions with similar health-system characteristics that are increasingly exposed to severe climate-related events.^30^

The clinical relevance of these disruptions may be greatest for patients with complex and continuous treatment needs, for whom even brief interruptions in access to controlled medicines may compromise symptom control and continuity of care.^3^^,^^4^^,^^11^^,^^12^ Although aggregate dispensing subsequently returned towards or intermittently exceeded expected levels, this does not establish that the same patients resumed treatment or that accumulated unmet needs were resolved. Recovery in overall dispensing volume should therefore not be equated with health-system resilience. A resilient system should be capable not only of restoring activity after a disaster but also of preserving access to essential treatments during the acute phase. Disaster preparedness should consequently include contingency stocks, alternative dispensing routes, emergency prescribing and authorisation mechanisms, interoperable health-information systems, and active identification and follow-up of patients whose treatment cannot be safely interrupted.^9^^,^^12^^,^^26^

Several limitations should be considered. First, SIA-SUS contains aggregated administrative records and does not provide information on clinical indications, prescribing decisions, medication consumption, adherence, individual treatment interruptions, or clinical outcomes.^19^, ^20^, ^21^^,^^28^ Second, the available data cannot distinguish genuine reductions in dispensing from delays, incomplete submission, or losses in administrative recording during the floods. Third, the mechanisms underlying differences across opioid agents and age groups could not be directly examined. Fourth, Minas Gerais provided a contextual comparison, but differences in baseline dispensing patterns, health-service organisation, and unmeasured time-varying factors between states cannot be excluded. Fifth, the available post-flood follow-up limits assessment of whether later monthly fluctuations represent sustained changes, temporary rebound effects, or a return to the pre-flood trajectory. Sixth, the state-level analysis may conceal substantial local heterogeneity according to flood severity, infrastructure damage, and health-service disruption. Seventh, the control model used for the Minas Gerais comparison showed weak statistical fit (adjusted R2 = 0.040) and strong residual autocorrelation (Durbin–Watson = 0.198), which limits the explanatory power of this model and constrains the strength of the comparison as evidence of regional specificity. Eighth, methadone dispensing in the control state showed an independent declining trend that does not align with the variable and imprecise estimates observed for methadone in Rio Grande do Sul, representing an additional confounding factor that should be considered when interpreting the agent-specific comparisons with the control state. Finally, the study captures outpatient procedures recorded within the public health system and does not include private-sector dispensing or medicines obtained through other pathways.

### Conclusion

May 2024 floods were associated with a marked and immediate reduction in recorded outpatient opioid dispensing in Rio Grande do Sul, followed by early stabilisation and an uneven restoration of activity. The magnitude and timing of the decline, together with the documented disruption of health services and transport infrastructure, are consistent with a major system-level shock that likely affected both dispensing and administrative reporting. Subsequent recovery in aggregate volumes does not necessarily imply that therapeutic continuity was restored for individual patients. Although context-specific, these findings may also inform preparedness in regions with similar health-system characteristics that have historically faced major climate-related emergencies less frequently but are increasingly exposed to them. Disaster-preparedness strategies should therefore prioritise the preservation of access to essential controlled medicines and the protection of patients with complex and continuous treatment needs during climate-related emergencies.

## Contributors

EF, FO and JNS conceived and designed the study. EF and JC performed data curation. EF, JC and JNS conducted statistical analyses. EF and JNS directly accessed and verified the underlying data reported in the manuscript. JNS independently reviewed the analytical dataset and verified the integrity and accuracy of the statistical outputs. SMD, FO, SH, and FK contributed to interpretation of the data and critically revised the manuscript for important intellectual content. All authors had full access to all the data in the study, contributed to drafting or revising the manuscript, approved the final version for submission, and accepted responsibility for the integrity of the data and the accuracy of the data analysis. The corresponding author confirms that all authors meet ICMJE criteria for authorship.

## Data sharing statement

Data collected for this study are publicly available from the Brazilian Unified Health System (Sistema Único de Saúde, SUS) through the Ambulatory Care Information System (SIA-SUS), hosted by the Department of Informatics of the SUS (DATASUS). The data used in this analysis consist of deidentified, administrative outpatient dispensing records aggregated at the monthly level. All data underlying the findings are accessible through the DATASUS public repositories (https://datasus.saude.gov.br/) and can be retrieved using the *microdatasus* R package. The analytic dataset and R scripts used for data cleaning, aggregation, and non-parametrictime-series modelling will be made available upon reasonable request to the corresponding author beginning at publication and with no end date. No individual-level identifiable participant data were used in this study. Additional materials, including the statistical code and model specifications, will be shared for academic and non-commercial research purposes upon request.

## Declaration of generative AI and AI-assisted technologies in the manuscript preparation process

During the preparation of this work, the authors used ChatGPT in order to improve language and formatting. After using this tool/service, the authors reviewed and edited the content as needed and take full responsibility for the content of the published article.

## Declaration of interests

FK has received personal fees as speaker and/or consultant from Janssen (J&J), Daiichi Sankyo, Libbs, Abbott, Eurofarma, and Teva Pharmaceutical Industries. The remaining authors declare no competing interests.
