# Supplementary material for: Outpatient opioid dispensing during the 2024 floods in Southern Brazil: a population-based ecological time-series analysis
Source: Lancet Reg Health Am. 2026 Sep 22;64:101646. doi: 10.1016/j.lana.2026.101646 (PMC13654047; doi:10.1016/j.lana.2026.101646)
Supplement: Supplementary file 1 [file mmc1.pdf]

Appendix: supplementary results for **"Outpatient Opioid Dispensing During the 2024 Floods in Southern Brazil: A Population-Based Ecological Time-Series Analysis"**

Enzo Fabian Frachia[a]

Juliana Vargas Campiol, BSc[a]

Santiago Madeira Diefenthaeler[b]

Felipe Ornell, PhD[b]

Simone Hauck, PhD[c]

Flavio Kapczinski, MD, PhD[b]

Juliana Nichterwitz Scherer, PhD[a]

[a]Programa de Pós-Graduação em Saúde Coletiva, Universidade do Vale do Rio dos Sinos (UNISINOS), São Leopoldo, RS, Brazil

[b]Universidade Federal do Rio Grande do Sul (UFRGS), Porto Alegre, RS, Brazil

[c]Programa de Pós-Graduação em Psiquiatria e Ciências do comportamento, Universidade Federal do Rio Grande do Sul (UFRGS), Porto Alegre, RS, Brazil

This document provides supplementary information that could not be included in the main article due to limitations on word count and the number of tables and figures. The supplementary appendix was submitted alongside the main manuscript to the journal and underwent peer review as part of the original submission.

Table of Contents

Section 1: Supplemental Tables 3

Supplementary Table 1. Descriptive characteristics of units outpatient opioid dispensations Minas Gerais, Brazil, January 2015 - December 2025 3

Supplementary Table 2. Non-Parametric Ecological Time-Series Analysis of Outpatient Opioid Dispensing After the May 2024 Flooding, Minas Gerais, Brasil 4

Supplementary Table 3. Sensitivity analysis excluding the COVID-19 period 5

Supplementary Table 4. Sensitivity Analysis Comparing Linear and Quadratic Time Trend Specifications. 6

Supplementary Table 5. Total number of dispensed opioids and population by year, Rio Grande do Sul, Brazil, 2015–2025 6

Section 2: Supplemental Figures 7

Supplementary Image 1. Observed and modeled total opioid dispensing rates and estimated monthly effects in Minas Gerais during the 2024 flood period (Control) 7

Supplementary Figure 2. Observed and modeled total opioid dispensing rates and estimated monthly effects following the 2024 floods, by active pharmaceutical ingredients in Minas Gerais (Control) 8

Section 1: Supplemental Tables

Supplementary Table 1. Descriptive characteristics of units outpatient opioid dispensations Minas Gerais, Brazil, January 2015 - December 2025.

Variable	Total - n (%)	Codeine - n (%)	Morphine - n (%)	Methadone - n (%)
**Total dispensation**	13,031,430 (100·0)	5,043,552 (38·7)	5,579,570 (42·8)	2,408,308 (18·5)
**Year**				
**2015**	1,128,612 (8·7)	423,483 (8·4)	520,803 (9·3)	184,326 (7·7)
**2016**	1,407,377 (10·8)	597,007 (11·8)	585,463 (10·5)	224,907 (9·3)
**2017**	1,361,615 (10·4)	576,706 (11·4)	589,304 (10·6)	195,605 (8·2)
**2018**	958,398 (7·4)	399,922 (7·9)	417,618 (7·5)	140,858 (5·8)
**2019**	316,255 (2·4)	132,712 (2·6)	119,271 (2·1)	64,272 (2·7)
**2020**	1,108,388 (8·5)	457,009 (9·1)	440,943 (7·9)	210,436 (8·7)
**2021**	1,303,515 (10·0)	493,175 (9·8)	566,480 (10·2)	243,860 (10·1)
**2022**	1,316,004 (10·1)	479,301 (9·5)	569,045 (10·2)	267,658 (11·1)
**2023**	1,149,505 (8·8)	442,998 (8·8)	428,551 (7·7)	277,956 (11·5)
**2024**	1,414,260 (10·9)	510,680 (10·2)	625,857 (11·2)	277,723 (11·5)
**2025**	1,567,501 (12·0)	530,559 (10·5)	716,235 (12·8)	320,707 (13·4)
**Age group (years)**				
**0-19**	89,869 (0·7)	7,669 (0·2)	68,576 (1·2)	13,624 (0·6)
**20-39**	1,331,055 (10·2)	384,614 (7·6)	645,861 (11·6)	300,580 (12·5)
**40-59**	6,224,034 (47·8)	2,341,482 (46·4)	2,570,882 (46·1)	1,311,670 (54·5)
**60-79**	4,748,524 (36·4)	1,975,984 (39·2)	2,051,222 (36·8)	721,318 (30·0)
**80+**	637,948 (4·9)	333,803 (6·6)	243,029 (4·4)	61,116 (2·4)
**Sex**				
**Female**	7,313,422 (56·1)	3,048,840 (60·5)	2,959,444 (53·0)	1,305,138 (54·2)
**Male**	5,718,008 (43·9)	1,994,712 (39·5)	2,620,126 (47·0)	1,103,170 (45·8)

Note: presented as absolute counts (n) and column percentages (%). For this descriptive analysis, percentages represent the proportion of dispensations within each category (Year, Age Group, or Sex) relative to the total for that specific opioid agent (column total

Supplementary Table 2. Non-Parametric Ecological Time-Series Analysis of Outpatient Opioid Dispensing After the May 2024 Flooding, Minas Gerais, Brasil

Variable	Flood month effect (May 2024) in dispensing rates (95% CI)	One-month post-flood effect (June 2024) in dispensing rates (95% CI)	Two-month post-flood effect (July 2024) in dispensing rates (95% CI)	Three-month post-flood effect (August 2024) in dispensing rates (95% CI)	Four-month post-flood effect (September 2024) in dispensing rates (95% CI)	Five-month post-flood effect (October 2024) in dispensing rates (95% CI)	Six-month post-flood effect (November 2024) in dispensing rates (95% CI)	Seven-month post-flood effect (December 2024) in dispensing rates (95% CI)	Eight-month post-flood effect (January 2025) in dispensing rates (95% CI)	Nine-month post-flood effect (February 2025) in dispensing rates (95% CI)	Ten-month post-flood effect (March 2025) in dispensing rates (95% CI)	Eleven-month post-flood effect (April 2025) in dispensing rates (95% CI)	Twelve-month post-flood effect (May 2025) in dispensing rates (95% CI)	Adjusted R²	Durbin-Watson test
**Total opioids**	0·3852 (-10·4987 to 11·2691)	-3·0979 (-13·6367 to 7·4409)	0·4853 (-10·2256 to 11·1963)	1·4234 (-9·1638 to 12·0106)	0·4961 (-11·7575 to 12·7496)	5·1429 (-5·6394 to 15·9253)	3·2238 (-5·5574 to 12·0049)	-3·7372 (-12·8371 to 5·3627)	3·0729 (-8·1062 to 14·2521)	0·9367 (-9·7300 to 11·6035)	-7·2643 (-18·6153 to 4·0866)	-2·1081 (-12·8179 to 8·6017)	-4·2677 (-16·8092 to 8·2739)	0·040	0·198
**By opioid agent**															
**Codeine**	0·4365 (-3·9240 to 4·7971)	-0·8319 (-5·1362 to 3·4723)	1·0983 (-3·0797 to 5·2763)	0·3931 (-3·2111 to 3·9973)	0·5467 (-3·8226 to 4·9160)	1·5837 (-2·4144 to 5·5818)	1·2236 (-2·3252 to 4·7725)	-1·3723 (-5·5741 to 2·8296)	2·2412 (-2·8042 to 7·2865)	0·4517 (-4·2577 to 5·1612)	-1·6344 (-6·3618 to 3·0929)	-0·7503 (-5·1776 to 3·6771)	-2·2974 (-7·4526 to 2·8578)	-0·089	0·264
**Morphine**	0·5951 (-4·7427 to 5·9329)	-1·4628 (-6·3787 to 3·4531)	3·1465 (-2·2567 to 8·5497)	5·7875 (-0·8617 to 12·4368)	1·7279 (-5·5329 to 8·9887)	4·2757 (-1·8191 to 10·3705)	2·8112 (-1·5286 to 7·1510)	-0·8664 (-5·1179 to 3·3852)	2·8730 (-2·7560 to 8·5021)	1·7394 (-3·3495 to 6·8284)	-2·4060 (-7·8499 to 3·0380)	0·7918 (-4·3903 to 5·9739)	0·5891 (-5·5664 to 6·7446)	0·088	0·279
**Methadone**	-0·6464 (-2·7054 to 1·4126)	-0·8032 (-2·8705 to 1·2640)	-3·7595 (-5·6944 to -1·8245)***	-4·7572 (-6·7576 to -2·7567)***	-1·7785 (-3·6823 to 0·1254)	-0·7164 (-2·5909 to 1·1580)	-0·8111 (-2·6801 to 1·0579)	-1·4986 (-3·4970 to 0·4999)	-2·0413 (-3·9264 to -0·1561)*	-1·2545 (-3·2521 to 0·7432)	-3·2240 (-5·3903 to -1·0576)**	-2·1496 (-4·3370 to 0·0378)	-2·5593 (-5·0374 to -0·0813)*	0·356	0·189
**By age group**															
**0-19 years**	0·2034 (0·0376 to 0·3692)*	0·2387 (0·1343 to 0·3430)***	0·1518 (0·0481 to 0·2555)**	0·0506 (-0·0763 to 0·1774)	0·0448 (-0·0655 to 0·1551)	0·4172 (0·2814 to 0·5530)***	0·2414 (0·0622 to 0·4205)**	0·0215 (-0·0765 to 0·1194)	0·2110 (0·0348 to 0·3871)*	-0·0054 (-0·1516 to 0·1407)	-0·0709 (-0·2606 to 0·1187)	0·0395 (-0·0902 to 0·1692)	-0·0398 (-0·2314 to 0·1518)	0·480	1·494
**20-39 years**	0·3210 (-0·7639 to 1·4059)	-0·1203 (-1·3955 to 1·1548)	-0·2739 (-1·4043 to 0·8565)	-0·2718 (-1·3326 to 0·7890)	0·5225 (-0·7473 to 1·7923)	0·7089 (-0·3427 to 1·7606)	1·0747 (0·1327 to 2·0167)*	0·8695 (-0·0348 to 1·7738)	0·3537 (-0·6740 to 1·3814)	0·8202 (-0·2273 to 1·8677)	-1·3009 (-2·3759 to -0·2260)*	-0·1397 (-1·2930 to 1·0135)	-0·2809 (-1·5118 to 0·9500)	-0·004	0·348
**40-59 years**	-0·0560 (-5·3045 to 5·1925)	-1·7695 (-6·9297 to 3·3907)	-0·6224 (-5·8734 to 4·6286)	-1·9662 (-6·9919 to 3·0595)	-1·3026 (-7·2639 to 4·6587)	1·0595 (-4·4684 to 6·5875)	0·6101 (-3·7862 to 5·0065)	-2·7661 (-7·2808 to 1·7487)	-0·1763 (-5·6340 to 5·2814)	0·7883 (-4·3466 to 5·9233)	-4·0928 (-9·7263 to 1·5407)	0·1906 (-4·9618 to 5·3430)	-2·1990 (-8·2677 to 3·8697)	-0·026	0·187
**60-79 years**	0·0137 (-4·0049 to 4·0323)	-1·4763 (-5·2573 to 2·3047)	1·2971 (-2·4925 to 5·0866)	2·9633 (-1·0551 to 6·9817)	0·6472 (-3·8748 to 5·1691)	2·3757 (-1·3896 to 6·1410)	0·7074 (-2·4789 to 3·8938)	-1·7053 (-4·9819 to 1·5712)	1·5760 (-2·5254 to 5·6775)	-0·4549 (-4·6015 to 3·6916)	-2·0709 (-6·2555 to 2·1136)	-1·9581 (-5·9537 to 2·0374)	-1·6467 (-6·2659 to 2·9725)	0·134	0·219
**80+ years**	-0·0968 (-0·7179 to 0·5243)	0·0296 (-0·5553 to 0·6144)	-0·0672 (-0·7190 to 0·5846)	0·6476 (0·0523 to 1·2428)*	0·5842 (-0·0388 to 1·2071)	0·5815 (0·0134 to 1·1497)*	0·5901 (0·1288 to 1·0514)*	-0·1568 (-0·7201 to 0·4066)	1·1085 (0·3809 to 1·8362)**	-0·2115 (-0·7776 to 0·3547)	0·2712 (-0·3064 to 0·8488)	-0·2404 (-0·8916 to 0·4109)	-0·1013 (-0·8295 to 0·6269)	0·143	0·454

Note: Estimates are presented as changes in dispensing rates (95% CI). Adjusted R² and Durbin–Watson statistics indicate model fit and residual autocorrelation, respectively. * (p<0·05); ** (p<0·01); *** (p<0·001).

Supplementary Table 3. Sensitivity analysis excluding the COVID-19 period

**Variable**	**Flood month effect (May 2024) in dispensing rates (95% CI)**	**One-month post-flood effect (June 2024) in dispensing rates (95% CI)**	**Two-month post-flood effect (July 2024) in dispensing rates (95% CI)**	**Three-month post-flood effect (August 2024) in dispensing rates (95% CI)**	**Four-month post-flood effect (September 2024) in dispensing rates (95% CI)**	**Five-month post-flood effect (October 2024) in dispensing rates (95% CI)**	**Six-month post-flood effect (November 2024) in dispensing rates (95% CI)**	**Seven-month post-flood effect (December 2024) in dispensing rates (95% CI)**	**Eight-month post-flood effect (January 2025) in dispensing rates (95% CI)**	**Nine-month post-flood effect (February 2025) in dispensing rates (95% CI)**	**Ten-month post-flood effect (March 2025) in dispensing rates (95% CI)**	**Eleven-month post-flood effect (April 2025) in dispensing rates (95% CI)**	**Twelve-month post-flood effect (May 2025) in dispensing rates (95% CI)**	**Adjusted R²**	**Durbin-Watson test**
**Total opioids**	-68·6994 (-71·8978 to -65·5010)***	-13·9746 (-18·8995 to -9·0497)***	-2·1000 (-8·1420 to 3·9419)	1·8854 (-1·4364 to 5·2071)	5·7277 (2·5578 to 8·8976)***	10·5245 (8·1334 to 12·9155)***	3·2340 (-0·3906 to 6·8586)	-0·1757 (-4·1395 to 3·7881)	2·5710 (-1·3990 to 6·5410)	-6·0328 (-9·8459 to -2·2196)**	-6·3896 (-10·8096 to -1·9696)**	4·0577 (-0·7901 to 8·9055)	1·6365 (-2·0588 to 5·3318)	0·950	0·837
**By opioid agent**															
**Codeine**	-37·5340 (-39·8497 to -35·2183)***	-2·1627 (-4·6305 to 0·3051)	-0·0998 (-2·6998 to 2·5002)	1·3222 (-0·0326 to 2·6771)	2·5920 (0·8559 to 4·3281)**	4·9964 (2·9171 to 7·0757)***	-0·7284 (-2·1553 to 0·6985)	-2·3726 (-4·7797 to 0·0345)	3·1226 (0·7405 to 5·5047)*	-1·5324 (-4·0266 to 0·9617)	-0·4045 (-3·3542 to 2·5453)	3·3758 (0·4853 to 6·2663)*	0·9927 (-1·6306 to 3·6161)	0·954	0·734
**Morphine**	-16·7179 (-17·7504 to -15·6854)***	-7·2727 (-8·5162 to -6·0291)***	-2·7105 (-3·9985 to -1·4225)***	-2·6185 (-3·7155 to -1·5215)***	0·1162 (-1·4728 to 1·7053)	1·4247 (0·5133 to 2·3362)**	1·8505 (0·5877 to 3·1133)**	3·2784 (1·9589 to 4·5978)***	1·1496 (-0·1194 to 2·4185)	0·6899 (-0·4527 to 1·8325)	-2·4920 (-3·7492 to -1·2349)***	0·1684 (-1·3306 to 1·6673)	-0·0432 (-1·2381 to 1·1518)	0·883	1·567
**Methadone**	-14·4475 (-15·3309 to -13·5642)***	-4·5393 (-6·5259 to -2·5526)***	0·7102 (-2·0466 to 3·4671)	3·1816 (1·1490 to 5·2142)**	3·0195 (1·7004 to 4·3385)***	4·1033 (2·6634 to 5·5433)***	2·1119 (0·1688 to 4·0550)*	-1·0814 (-2·2911 to 0·1283)	-1·7011 (-3·1329 to -0·2693)*	-5·1902 (-6·6111 to -3·7693)***	-3·4931 (-5·0396 to -1·9466)***	0·5135 (-1·4177 to 2·4447)	0·6869 (-0·6338 to 2·0075)	0·836	0·743
**By age group**															
**0-19 years**	-0·1725 (-0·2600 to -0·0851)***	-0·0503 (-0·1036 to 0·0031)	0·0919 (0·0302 to 0·1535)**	-0·0757 (-0·1781 to 0·0268)	-0·0838 (-0·1618 to -0·0057)*	-0·0718 (-0·1624 to 0·0187)	-0·0667 (-0·1626 to 0·0292)	-0·0577 (-0·1329 to 0·0174)	-0·0676 (-0·1505 to 0·0153)	-0·1319 (-0·2118 to -0·0520)**	-0·0505 (-0·1418 to 0·0409)	0·0845 (-0·0332 to 0·2023)	-0·0536 (-0·1679 to 0·0607)	0·096	0·812
**20-39 years**	-6·4717 (-7·0676 to -5·8759)***	-2·4095 (-3·1800 to -1·6390)***	-1·2044 (-2·1473 to -0·2616)*	0·6560 (-0·0103 to 1·3222)	0·8056 (0·1489 to 1·4624)*	1·2581 (0·6557 to 1·8605)***	-0·2227 (-0·8735 to 0·4280)	-0·0339 (-0·6110 to 0·5432)	0·3602 (-0·2187 to 0·9391)	-0·6542 (-1·4081 to 0·0996)	-0·4013 (-1·1280 to 0·3255)	-0·2091 (-1·2610 to 0·8428)	0·0843 (-0·6822 to 0·8508)	0·829	1·133
**40-59 years**	-36·0849 (-37·5558 to -34·6139)***	-7·3011 (-9·8550 to -4·7471)***	-0·4173 (-3·2315 to 2·3969)	2·1883 (0·4092 to 3·9674)*	2·1003 (0·4521 to 3·7486)*	5·5456 (4·2317 to 6·8595)***	0·9642 (-1·2164 to 3·1448)	-0·8710 (-2·9940 to 1·2519)	1·0609 (-1·0055 to 3·1274)	-3·8317 (-5·8823 to -1·7812)***	-3·4976 (-5·8320 to -1·1631)**	0·4967 (-2·2452 to 3·2386)	-0·5036 (-2·4132 to 1·4060)	0·951	0·903
**60-79 years**	-23·5790 (-25·3943 to -21·7637)***	-4·1758 (-6·2575 to -2·0940)***	-0·3360 (-2·9175 to 2·2456)	-0·4647 (-1·7403 to 0·8110)	2·4328 (1·3114 to 3·5542)***	3·8652 (2·7630 to 4·9673)***	2·8144 (1·6303 to 3·9984)***	0·5648 (-0·8810 to 2·0106)	1·5295 (-0·1154 to 3·1744)	-1·4115 (-2·9663 to 0·1433)	-1·8659 (-3·8157 to 0·0840)	3·2797 (1·5305 to 5·0289)***	2·0821 (0·2411 to 3·9231)*	0·940	0·699
**80+ years**	-2·3913 (-2·9438 to -1·8388)***	-0·0379 (-0·4138 to 0·3379)	-0·2342 (-0·5561 to 0·0877)	-0·4186 (-0·8966 to 0·0595)	0·4727 (-0·0117 to 0·9571)	-0·0726 (-0·5921 to 0·4468)	-0·2552 (-0·7323 to 0·2218)	0·2222 (-0·4164 to 0·8609)	-0·3120 (-0·8533 to 0·2293)	-0·0035 (-0·5593 to 0·5524)	-0·5744 (-1·1650 to 0·0161)	0·4059 (-0·1864 to 0·9981)	0·0273 (-0·6626 to 0·7171)	0·719	0·647

Note: Estimates are presented as changes in dispensing rates (95% CI). Adjusted R² and Durbin–Watson statistics indicate model fit and residual autocorrelation, respectively. * (p<0·05); ** (p<0·01); *** (p<0·001)

Supplementary Table 4. Sensitivity Analysis Comparing Linear and Quadratic Time Trend Specifications.

Variable	ΔAIC	ΔBIC	Adjusted R² (Linear)	Adjusted R² (Quadratic)	Preferred model
**Total**	11·17	8·3	0·9294	0·9355	Quadratic
**Codeine**	15·59	12·71	0·9406	0·9476	Quadratic
**Morphine**	5·7	2·83	0·8224	0·8309	Quadratic
**Methadone**	-0·52	-3·39	0·8221	0·8224	Inconclusive*
**0-19**	18·13	15·25	-0·0637	0·079	Quadratic
**20-39**	3·91	1·04	0·8008	0·8077	Inconclusive*
**40-59**	13·09	10·22	0·9252	0·9327	Quadratic
**60-79**	19·54	16·66	0·9099	0·9228	Quadratic
**80+**	2·43	-0·45	0·7487	0·7547	Inconclusive*

Note: ΔAIC and ΔBIC represent the absolute difference between the values of the Akaike Information Criterion (AIC) and Bayesian Information Criterion (BIC) obtained for the linear and quadratic models (ΔAIC = (AIClinear − AICquadratic); ΔBIC = (BIClinear − BICquadratic)). Lower values indicate similar fit between the specifications.*Inconclusive: Differences in AIC and BIC were small (<4 units) and/or the two criteria favored different model specifications. Therefore, neither the linear nor the quadratic model demonstrated a clear advantage.

Supplementary Table 5. Total number of dispensed opioids and population by year, Rio Grande do Sul, Brazil, 2015–2025

Year	Prescription of opioid	Population
2015	197,079	11,247,972
2016	276,198	11,286,500
2017	386,539	11,322,895
2018	404,518	11,329,605
2019	478,437	11,377,239
2020	623,222	11,422,973
2021	684,550	11,466,630
2022	723,338	11,507,906
2023	792,029	11,546,505
2024	894,075	11,582,285
2025	1,112,853	11.233.263

Note: Annual counts of opioid prescriptions and corresponding population estimates are presented for each year. Prescription data were obtained from SIA-SUS/DATASUS (2015–2025), and population estimates were obtained from the Instituto Brasileiro de Geografia e Estatística (IBGE)

Section 2: Supplemental Figures

Supplementary Image 1. Observed and modeled total opioid dispensing rates and estimated monthly effects in Minas Gerais during the 2024 flood period (Control)

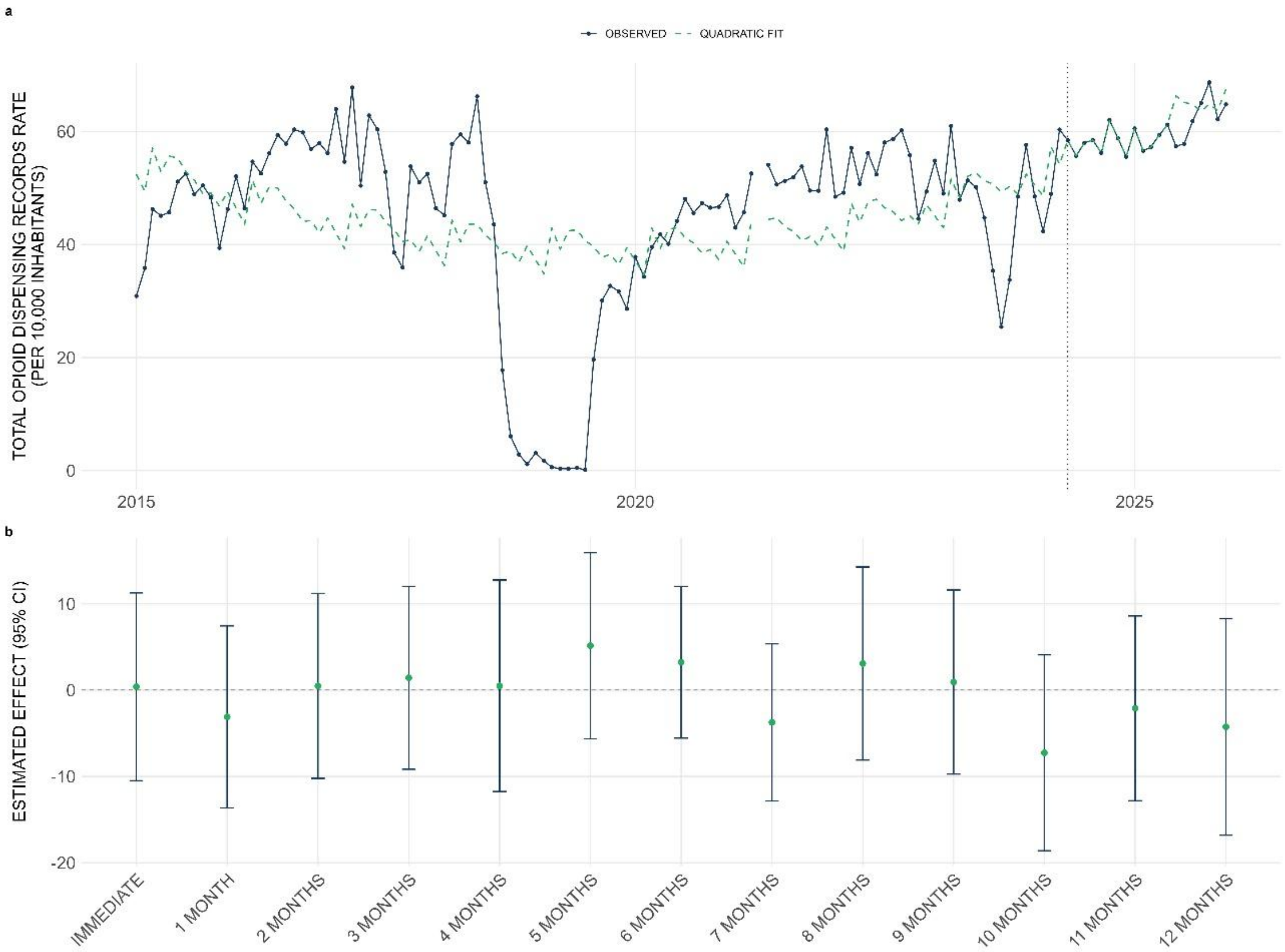

Note: (a) Observed monthly total opioid dispensing rate (per 10,000 inhabitants) with quadratic trend fit, 2015–2025, for Minas Gerais (control state); the vertical dotted line indicates the corresponding flood period in Rio Grande do Sul (May 2024). (b) Estimated monthly effects on dispensing rate (95% CI) relative to the counterfactual trend, from immediate to 12 months following the corresponding flood period.

Supplementary Figure 2. Observed and modeled total opioid dispensing rates and estimated monthly effects following the 2024 floods, by active pharmaceutical ingredients in Minas Gerais (Control)

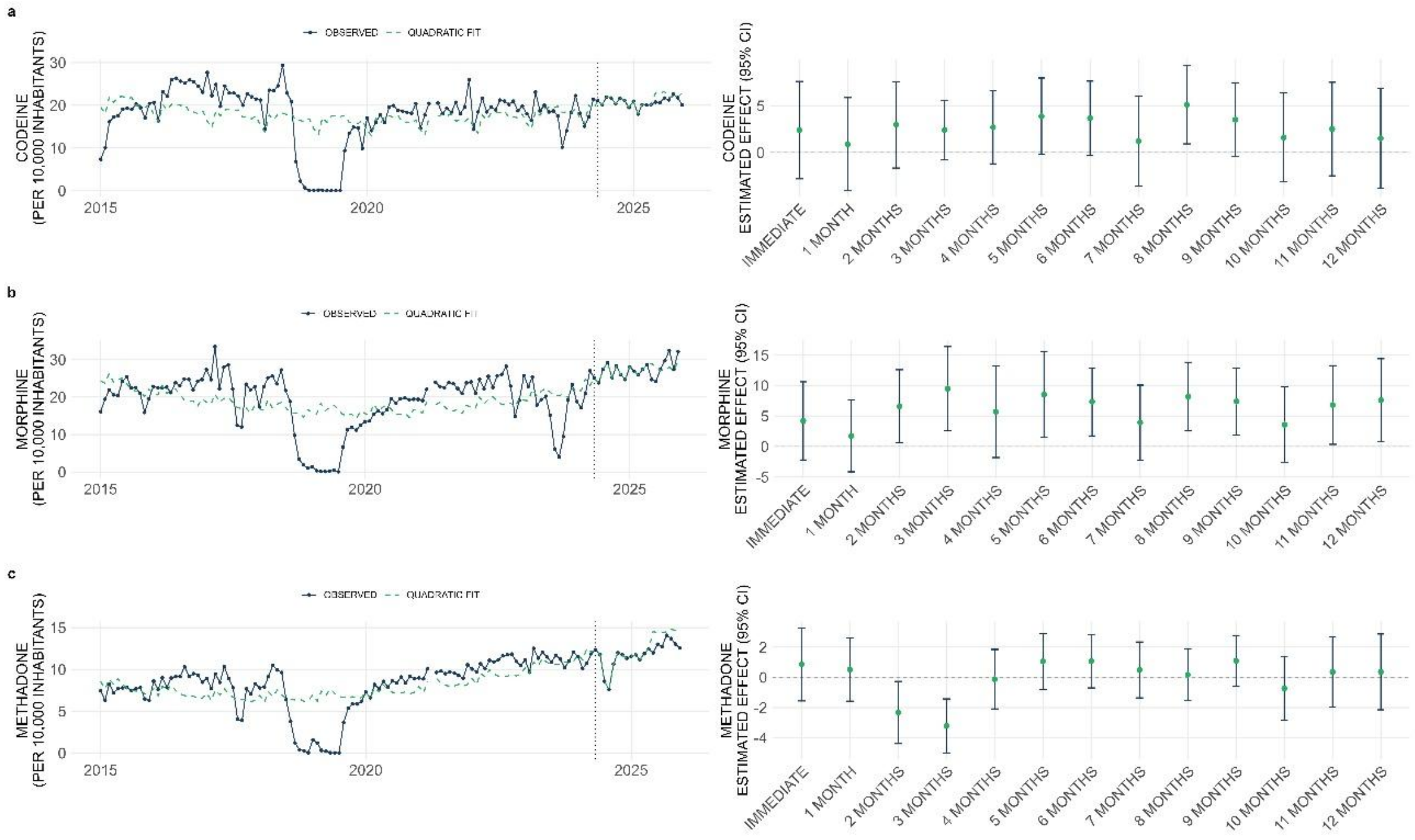

Note: Monthly opioid units dispensed (per 10,000 inhabitants) with quadratic trend fit for (a) codeine, (b) morphine, and (c) methadone. Observed monthly total opioid dispensing rate (per 10,000 inhabitants) with quadratic trend fit, 2015–2025, for Minas Gerais (control state); the vertical dotted line indicates the corresponding flood period in Rio Grande do Sul (May 2024). (b) Estimated monthly effects on dispensing rate (95% CI) relative to the counterfactual trend, from immediate to 12 months following the corresponding flood period.
